# Testing the validity of the attention control video: An eye-tracking approach of the ego depletion effect

**DOI:** 10.1371/journal.pone.0211181

**Published:** 2019-01-22

**Authors:** Chris Englert, Dennis Koroma, Alex Bertrams, Corinna S. Martarelli

**Affiliations:** 1 Institute of Educational Science, University of Bern, Bern, Switzerland; 2 Institute for Sport Sciences, Goethe University Frankfurt, Frankfurt, Germany; 3 Institute of Psychology, University of Bern, Bern, Switzerland; 4 Swiss Distance Learning University, Brig, Switzerland; SWPS University of Social Sciences and Humanities, POLAND

## Abstract

The attention control video has been frequently applied to test the ego depletion effect. However, its validity has never been tested, a shortcoming we address in this preregistered study. In the first task, self-control strength was temporarily depleted in the depletion condition (*n* = 56) but remained intact in the control condition (*n* = 56). The attention control video served as the secondary task, and we assumed that the depletion condition would perform significantly worse compared to the control condition. Attention regulation was measured with an eye-tracking device. The results revealed that the gaze behavior in the two conditions differed statistically significantly; however, the actual difference was small, indicating that the attention control video may not be an optimal measure of self-control.

## Introduction

Volitionally controlling one’s attention, impulses, emotions, or thoughts can be difficult and cannot always be accomplished, as self-control does not always work [1]. The strength model posits that all different facets of self-control are based on one metaphorical global self-control energy resource with limited capacity [2]. Individuals’ self-control strength can become temporarily depleted after they work on a self-control task. In a state of *ego depletion*, subsequent self-control performance suffers [3].

The *sequential two-task paradigm* is the most common research approach for investigating the ego depletion effect [3]. Participants are randomly assigned to a depletion condition or a control condition and work on a similar first task. For instance, while watching a funny video clip participants are instructed to either suppress their emotions (the depletion condition) or not suppress their emotions (the control condition) [4]. As emotion suppression is considered a self-control act, it should lead to a state of ego depletion in the depletion condition while individuals’ self-control strength in the control condition should remain intact. It is assumed that depleted participants should show lower performance in a subsequent self-control task compared to participants in the control condition with still available resources. The assumptions of the strength model have been supported by numerous studies [5,6].

However, recent rigorous meta-analyses suggest the ego depletion effect is close to zero on average [7–8] and several researchers were not able to replicate the ego depletion effect [9–10]. Especially, the null findings of a recent Preregistered Replication Report [11] and a recent survey among ego depletion researchers [12] raise questions regarding the validity of the strength model. One major issue regarding the strength model is the application of mostly un-validated self-control tasks [12]. More precisely, it is unclear which tasks require self-control and which tasks do not. For instance, the null findings of the Preregistered Replication Report [11] were questioned about the appropriateness of the applied self-control tasks [13]. In general, “self-control is the exertion of control over the self by the self. That is, self-control occurs when a person (or other organism) attempts to change the way he or she would otherwise think, feel, or behave” [4] (p. 247). Tasks that match this definition of self-control include the transcription task [14], which is explained in more detail in the Method section, the Stroop task [15], and the attention control video [16]. In all of these tasks, individuals have to force themselves to suppress their dominant behaviors and perform a different behavior instead (e.g., inhibit their dominant word reading tendency and name the color in which a word is written instead) [15]. In the present study, we aim to test the validity of the attention control video [16] which has been frequently adopted in self-control research [17–20].

The attention control video is a 6-min mute video clip displaying a woman being interviewed by an off-camera interview partner. In the lower right corner of the screen, a series of common words appears (e.g., house) for 10 sec each. Participants in the depletion condition are instructed to ignore these words and to pay attention only to the woman, while participants in the control condition do not receive any instructions about how to watch the video. Researchers have demonstrated that stimuli that suddenly pop up in one’s visual periphery automatically draw attention [21]. This implies that self-control needs to be invested to volitionally override the automatic tendency to peek at the words in the lower right corner [22]. Therefore, in the depletion condition participants’ self-control strength should become depleted while it should remain intact in the control condition after participants watch the video clip.

However, there are two methodological flaws regarding the attention control video. First, as far as we are aware, whether participants truly follow the instructions and try to ignore the words in the lower right corner has never been measured. Second, and most important, a recent meta-analysis did not find a statistically significant effect of the attention control video on ego depletion [5].

One valid measure of attention regulation is the measurement of one’s gaze behavior, which can be achieved by applying eye-tracking devices [23]. Eye tracking can provide additional information over and above explicit responses. Eye position measures are continuous, online, and more implicit (i.e., less susceptible to desirable responses) than, for example, self-report measures. More precisely, in the context of this study, eye tracking assesses straightforwardly where overt attention is directed.

The aim of the present study was to test the validity of the attention control video in a preregistered experiment (https://aspredicted.org/zv6sd.pdf). Following the sequential two-task paradigm, self-control strength was first experimentally manipulated with an initial self-control task. Afterward, all participants watched the attention control video and were instructed to not pay any attention to the words and focus only on the woman being interviewed [16]. Self-control performance was measured by the percentage of the number of fixations on the woman being interviewed (i.e., the proportion of correct responses according to the instructions). In line with Schmeichel and colleagues [16], we assumed that ego-depleted participants would perform worse than participants with intact self-control strength.

## Materials and methods

### Ethics statement

This study has been approved by the local ethics committee. The study was carried out in accordance with the Declaration of Helsinki and the ethical guidelines for experimental research with human participants as proposed by the German Psychological Society (DGPs) and the American Psychological Association (APA). All persons gave their written informed consent prior to their inclusion in the study.

### Participants

An a priori G*Power analysis revealed that we needed a sample of 128 participants to detect at least a medium effect (parameters: *d* = 0.50, α = 0.05, 1−β = 0.80) [24]. 130 participants took part in the study. Two participants had to be excluded because they did not follow the instructions of the first self-control task. Sixteen additional participants had to be excluded due to technical problems in the eye tracking phase (13 had error values above 0.8° in the calibration and validation procedure, and three participants had a tracking ratio below 35%). The final sample consisted of 112 university students (*M*_age_ = 24.77, *SD*_age_ = 10.12; 87 female).

All participants had normal or corrected-to-normal visual acuity and were German speaking. They were naïve about the purpose of the study and received course credit for participation.

### Procedure

We chose a one-factor between-subjects design, as participants were randomly assigned to the depletion condition (*n* = 56) or the control condition (*n* = 56). The study was conducted in single sessions in a lab room with constant light conditions at the university. All questionnaires were administered on a computer screen, and higher overall scores on all of the questionnaires always indicate higher values of the respective variable (S1 File; for full protocol, see also dx.doi.org/10.17504/protocols.io.uj3euqn).

After providing demographic information (age, sex, and nationality), participants worked on the German short version of the Self-Control Scale (SCS-K-D) [25]. The SCS-K-D measures individuals’ general ability to regulate themselves (i.e., trait self-control), and we assumed that based on our randomization the conditions would not differ statistically significantly in this measure. The SCS-K-D consists of 13 items (e.g., “I am good at resisting temptations”; α = .787) which need to be answered on 5-point Likert-type scales (1 = *Not at all* to 5 = *Very much*).

In the next step, we manipulated the respondents’ self-control strength by using the transcription task (Bertrams et al., 2010). This task has been successfully applied several times and in different labs [26–29]. Participants were instructed to transcribe a neutral text on a separate sheet of paper by hand. In the depletion condition, participants were asked to omit the frequent letters “e” and “n” while transcribing the text, which requires self-control, as individuals have to force themselves to change their well-elaborated writing habits. In the control condition, participants transcribed the text conventionally, which should not have required a considerable amount of self-control strength. Contrary to our preregistration, we also manipulated the presentation of the transcription task. Participants were instructed either to transcribe as many words as possible for 6 min (*n* = 58) or to transcribe one paragraph (i.e., a fixed number of 134 words; *n* = 54). Therefore, the final sample included 29 participants in the depleted and fixed length time condition, 29 in the depleted and fixed number of words condition, 27 in the not-depleted and fixed length time condition, and 27 in the not-depleted and fixed number of words condition. By choosing this approach, we were able to test whether the results depended on a very specific presentation of the initial self-control task, which would limit their validity. Note that both types of presentation require the exertion of self-control; however, they differ in terms of where their standardization takes place (fixed length of time vs. fixed number of words). In contrast to previous applications of the transcription task (i.e., fixed length of time), fixing the number of words to be transcribed equalizes the number of responses that have to be overridden (i.e., self-control) across all participants.

To analyze whether the experimental manipulation of self-control strength was successful, a four-item manipulation check followed (e.g., “How strongly did you have to regulate your writing habits?”; α = .668) [14]. We expected the depletion condition to have higher scores on the four-item manipulation check. Participants responded to these items on 4-point Likert-type scales (1 = *Not at all* to 4 = *Very much*). To check whether the two different types of the transcription task (i.e., 6 min vs. fixed number of words that needed to be transcribed) were suited to manipulate self-control strength, we also compared the manipulation check scores between these two types of transcription task.

Next, we measured participants’ mood with the German version of the Positive and Negative Affect Schedule (PANAS) [30] to rule out the possibility that the transcription task led to unintended group differences in mood. Participants’ negative (e.g., “worried”; α = .655) and positive (e.g., “proud”; α = .872) moods were assessed with 10 items each. Each item was answered on a 5-point Likert-type scale (1 = *Not at all* to 5 = *Very much*). We also analyzed whether the two different types of transcription task (i.e., 6 min vs. fixed number of words that needed to be transcribed) affected mood differently.

Then, participants were informed that they would now watch a short video clip with no audio showing a woman who is being interviewed by a non-visible interviewer [16].

All participants were told to pay attention only to the woman and to ignore the words appearing in the lower right corner of the screen. If the participants happened to look at a word, they were asked to shift their focus back to the woman immediately. These instructions appeared on screen and were given only once before starting the task. As previously mentioned, this task requires self-control as individuals need to volitionally regulate their attention [21]. In line with Schmeichel and colleagues’ approach [16], participants were also informed that they would be asked a series of questions regarding the woman’s nonverbal behavior afterward to keep up the cover story. In the video phase of the study, eye movements were recorded using an SMI RED tracking system (SensoMotoric Instruments, Teltow, Germany). Data were registered at a sampling rate of 500 Hz, a spatial resolution of 0.1°, and a gaze position accuracy of 0.5°. The eye-tracking device was contact-free and determined the direction of gaze by combining the cornea reflex with the pupil location via an infrared light-sensitive video camera. The video was presented on a 22- inch screen (1280 × 1024 pixels) using SMI Experiment Center software, and eye data were recorded with I-View X software, both developed by SensoMotoric Instruments (Teltow, Germany). Participants were seated in front of a computer screen. The distance between participant and screen was approximately 70 cm. We used a 5-point calibration and validation procedure (only error values below 0.8° were accepted). The eye data analyses were based on fixations extracted using BeGazeTM software (SensoMotoric Instruments, Teltow, Germany). Fixations were detected when the sum of the gaze stream on the x- and y-axes was within an area of 100 pixels and when the fixation duration exceeded 80 ms. Blink events were automatically subtracted from the original gaze stream by the software and treated as missing data. We calculated the percentage of the number of fixations on the video frame (woman area of interest, AOI) as dependent variable. Proportions were based on fixations on the woman AOI divided by the sum of fixations to the screen.

After watching the video, participants answered six questions regarding the nonverbal behavior of the interviewed woman (e.g., “How attentive did the woman seem to be?”). As these items were used only to maintain the cover story, we did not run any analyses on these items. We also asked participants to indicate how difficult it was for them to suppress shifts of attention to the words and how much they had to force themselves to not pay attention to these words [16]. These two items were added to analyze whether the attention control video required self-control. All of these items were answered on 7-point Likert-type scales (1 = *Not at all* to 7 = *Very much*). Finally, participants were asked to guess the hypothesis of the study, thanked for their participation, and debriefed.

## Results

We conducted analyses of variance and report *p*-values, confidence intervals, and partial eta squared as measure of effect size (for full data set, see https://figshare.com/articles/Data_set/7203029). To quantify how much the data should shift our belief in favor of the null or the alternative hypothesis, we computed Bayes Factors (BF10 where 1 means that the two hypotheses are equally likely, larger values indicate more evidence for the alternative hypothesis, and smaller values indicate more evidence for the null hypothesis). All Bayesian analyses were computed with JASP version 0.8.6.

### Preliminary analyses

First, we performed preliminary analyses and ran 2 × 2 analyses of variances (ANOVAs) with condition (depletion vs. control) and type of standardization of the transcription task (fixed length of time vs. fixed number of words) as independent variables. Descriptive statistics are reported in Table 1 (see also S2 File).

**Table 1:** Descriptive statistics: Means and Standard Deviations.

	Experimental condition			
Variable	Depletion		Control	
	*M*	*SD*	*M*	*SD*
SCS-K-D	3.15	0.51	3.26	0.08
Manipulation Check Transcription task	2.38	0.50	1.74	0.41
PANAS positive	2.91	0.69	2.74	0.70
PANAS negative	1.31	0.27	1.23	0.26
Report on gaze behavior Item 1	4.75	1.78	4.95	1.57
Report on gaze behavior Item 2	4.63	1.51	4.50	1.44

*Note*. *n* = 56 in depletion condition, *n* = 56 in control condition. SCS-K-D = German short version of the Self-Control Scale. Manipulation Check Transcription Task = four-item manipulation check (e.g., “How strongly did you have to regulate your writing habits?”). PANAS positive = German version of the Positive and Negative Affect Schedule—positive affect. PANAS negative = German version of the Positive and Negative Affect Schedule—negative affect. Report on gaze behavior Item 1 = “How strongly did you have to force yourself to not pay attention to the words”. Report on gaze behavior Item 2 = “How difficult was it to not pay any attention to the words?”

#### SCS-K-D

The interaction between condition and type of standardization of the transcription task was not significant (*F*(1, 104) = 1.49, *p* = .224, η^2^_p_ = .014, BF10 = 0.538) nor was the main effect of condition (*F*(1, 104) = 1.41, *p* = .237, η^2^_p_ = .013, BF10 = 0.347). However, the main effect of type of standardization of the transcription task was marginally significant (*F*(1, 104) = 3.81, *p* = .054, η^2^_p_ = .035, BF10 = 1.094) such that participants in the fixed length of time condition (*M* = 3.30, *SE* = 0.07, 95% confidence interval (CI) = [3.16, 3.44]) reported a higher general ability to apply self-control compared to participants in the fixed number of words condition (*M* = 3.10, *SE* = 0.07, 95% CI = [2.95, 3.25]).

#### Manipulation check transcription task

To determine whether our manipulation of ego depletion was efficient we analyzed the answers to the four manipulation-check items. The dependent variable was the average of the four items. Participants in the depletion condition (*M* = 2.38, *SE* = 0.06, 95% CI = [2.25, 2.50]) had statistically significant higher values on the manipulation check (*F*(1, 108) = 54.11, *p* < .001, η^2^_p_ = .334, BF10 = 2.454e+8) than participants in the control condition (*M* = 1.74, *SE* = 0.07, 95% CI = [1.61, 1.86]). This indicates that our manipulation was successful. The main effect of standardization of the transcription task and the interaction yielded results that were not statistically significant (*Fs* < 1, BFs10< 0.303).

#### PANAS positive

There was no difference in positive mood between the groups. The main effect of condition (*F*(1, 108) = 1.72, *p* = .192, η^2^_p_ = .016, BF10 = 0.438), the main effect of standardization of the transcription task (*F* < 1, BF10 = 0.212), and the interaction (*F* < 1, BF10 = 0.262) yielded results that were not statistically significant.

#### PANAS negative

Similarly, there was no difference in negative mood between the groups. The main effect of condition (*F*(1, 108) = 2.98, *p* = .087, η^2^_p_ = .027, BF10 = 0.677), the main effect of standardization of the transcription task (*F* < 1, BF10 = 0.246), and the interaction (*F*(1, 108) = 3.23, *p* = .075, η^2^_p_ = .029, BF10 = 0.974) yielded results that were not statistically significant. Our manipulation did not lead to unintended group differences in mood state.

### Main analyses

Gaze position was analyzed in the period starting from the first appearance of the woman in the video until completion of the video (6 min). The video frame quadrant was defined as AOI, and we computed percentages of number of fixations on the woman AOI. The number of fixations on the woman AOI was divided by the total number of fixations on the screen. The independent-samples *t*-test revealed that participants in the depletion condition (*M* = 99.56, *SD* = 0.86) allocated statistically significantly fewer fixations on the woman AOI compared to participants in the control condition (*M* = 99.85, *SD* = 0.41), *t*(110) = 2.25, *p* = .026, *d* = 0.43, mean difference = 0.29, 95% CI = [0.03, 0.54], BF10 = 1.906. This effect remained statistically significant when controlling for standardization of the transcription task and positive and negative mood in an analysis of covariance (ANCOVA: *F*(1, 107) = 5.87, *p* = .017, η^2^_p_ = .052, BF10 = 1.906). Standardization of the transcription task (*F* < 1, BF10 = 0.219) and positive (*F*(1, 107) = 2.00, *p* = .160, η^2^_p_ = .018, BF10 = 0.345) and negative mood (*F* < 1, BF10 = 0.201) were not statistically significant.

To assure that the significant result we found with frequentist statistics is not a statistical artefact, we carried out a non-parametric test on the data. The Mann-Whitney U test confirmed our findings, *U* = 1252, *p* = 0.027. In addition, we computed the same independent *t*-test with arcsine-square-root transformed proportions (this transformation normalizes proportional data). We found the same significant difference, *t*(110) = 2.44, *p* = .016.

#### Time course analysis of the ego depletion effect

For the previous analysis, we considered the overall values of gaze behavior. However, the exact time interval of the depletion state has never been specified. To capture the temporal aspects of the ego depletion effect, we computed the number of fixations (percentages) on the woman AOI separately for 1-min time intervals and created the variable time interval (1 = fixations that were initiated in the time window between the occurrence of the video and 1 min after the onset of the video; 2 = fixations between 1 and 2 min; 3 = fixations between 2 and 3 min; 4 = fixations between 3 and 4 min; 5 = fixations between 4 and 5 min; and 6 = fixations between 5 and 6 min). An analysis of covariance with the variables time interval as repeated measurement factor (1, 2, 3, 4, 5, and 6) and condition as between-subjects factor (depletion vs. control) was computed for the number of fixations (percentages) on the woman AOI. We controlled for the standardization type of the transcription task and positive and negative mood.

**Fig 1:**
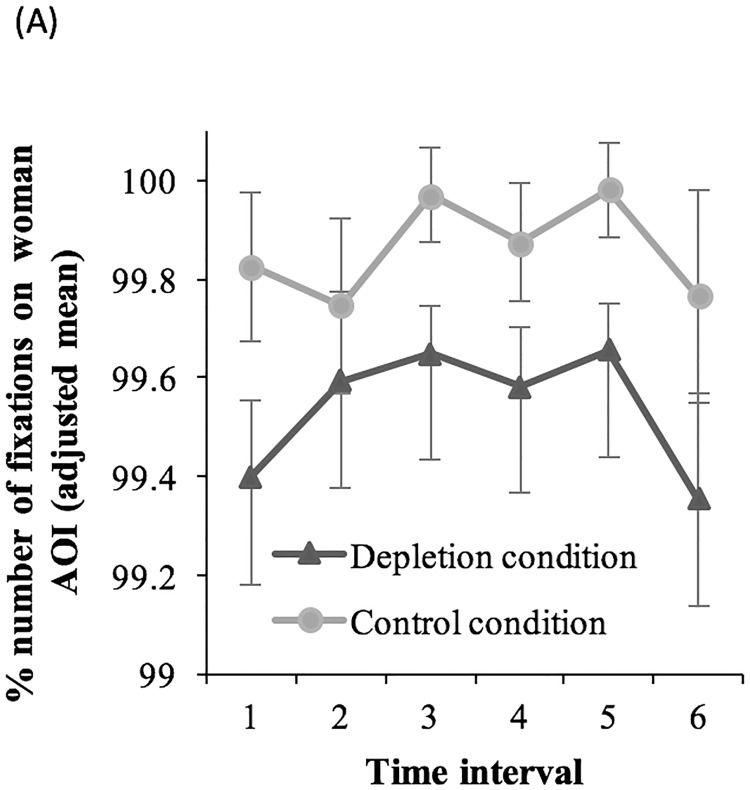
Time course analysis of the ego depletion effect. Adjusted mean percentage of the number of fixations during 0–1 min (interval 1), 1–2 min (interval 2), 2–3 min (interval 3), 3–4 min (interval 4), 4–5 min (interval 5), and 5–6 min (interval 6) for the two conditions (depletion vs. control). Covariates are the presentation type of the transcription task and positive and negative mood. Error bars indicate 1 standard error of the mean (SEM).

The analysis revealed a statistically significant difference between the depletion and control conditions, *F*(1, 106) = 5.59, *p* = .020, η^2^_p_ = .050, BF10 = 1.294. In the depletion condition (*M*_adjusted_ = 99.54%, *SE* = 0.10, 95% CI = [99.35, 99.73]), participants showed statistically significantly fewer fixations on the woman AOI compared to participants in the control condition (*M*_adjusted_ = 99.9%, *SE* = 0.10, 95% CI = [99.67, 100]). The variable time interval was not statistically significant, as well as the interaction condition by time interval (see Fig 1; *Fs* < 1, BFs10< 0.02).

#### Report on gaze behavior

Finally, we analyzed the two additional items on self-control following the attention control video, as participants had to indicate how difficult it was for them to suppress shifting their attention to the words and how much they had to force themselves to not pay attention to the words [16]. The answers to these two items were analyzed with two condition (depletion vs. control) × standardization type of the transcription task (fixed length of time vs. fixed number of words) ANOVAs. For the first question (“How strongly did you have to force yourself to not pay attention to the words”), we found no statistically significant results: main effect standardization of the transcription task, (*F*(1, 108) = 3.21, *p* = .076, η^2^_p_ = .029, BF10 = 0.857), main effect condition (*F* < 1, BF10 = 0.238), and interaction (*F* < 1, BF10 = 0.262). For the second question (i.e., “How difficult was it to not pay any attention to the words?”), we did not find any statistically significant differences either (*F*s < 1, BFs10<0.384).

## Discussion

Recent studies questioned the validity of the strength model [7–12] which led to a scientific debate regarding the magnitude of the ego depletion effect [13,31] and motivated us to analyze the validity of the attention control video [16]. The attention control video has often been used in ego depletion research [17–20]. It is postulated that self-control strength needs to be invested in order to ignore certain stimuli [16]. Therefore, we applied the sequential two-task paradigm [3]: In the first step, participants were either depleted or not depleted, while the attention control video was used as the dependent variable. We expected that depleted participants would be less adept at looking at a target stimulus in the presence of distracting stimuli than participants in the control condition.

The results partially support this assumption as the eye data analysis revealed that participants in the depletion condition allocated fewer fixations on the woman than participants in the control condition. However, the depleted and non-depleted participants were generally highly compliant with the task and most of the time fixated on the woman (more than 99% of fixations on the video). In addition, the actual mean differences that we found were very small (0.29%). Given the low variance in the dependent variable in both experimental conditions, even such a small difference could become statistically significant. However, is such a small difference relevant beyond statistical significance? The minimal differences in the means between the experimental conditions seem to indicate that the attention control video is not an appropriate self-control task. This result is in line with Dang’s [5] meta-analysis, which did not find a statistically significant effect of the attention control video on the subsequent self-control performance.. Recent work [32] calls for careful thinking about preregistered replication and bias-correcting meta-analytic procedures. The authors propose a Bayesian approach that takes into account the degree of belief in previous results and allows for updating prior beliefs (according to Bayes rule). Importantly, the method highlights the importance of considering practical significance. When it comes to the ego depletion effect, Carter and McCullough illustrate that the ego depletion effect approaches zero, which is consistent with our findings. The small difference we found turned out to be statistical significant but is practically insignificant.

In general, it seems promising to combine analyses of explicit behavioral responses with eye movements (which have voluntary and involuntary components) to get a deeper understanding of the ego depletion effect. The advantage of measuring eye movements, which is a non-invasive method, is that it gives an online and continuous measure of specific cognitive processes. This approach seems especially important, as there were no statistically significant group differences on the two self-report items on self-control following the attention control video. Therefore, although individuals did not have the impression that it was especially difficult for them to follow the instructions on an explicit level, there were tiny but measurable actual differences on the behavioral level.

The strengths of this study include the novelty of the eye-tracking approach in order to shed light on the underlying mechanisms of the ego depletion effect. With an explorative analysis, we investigated the time course of the ego depletion effect. A time course analysis might be a promising approach for exploring the exact duration of the ego depletion state which has not yet been specified. Therefore, we recommend an analysis of self-control performance over time when the attention control video is adopted in future studies on ego depletion [33].

A limitation of our study that needs to be considered is that the absence of eye movements does not imply the absence of covert shifts of attention. There is a strong relation between eye movements and attention, however they can be separated (overt vs. covert shifts of attention) [34]. It is possible that covert shifts of attention might vary to a greater extent than overt eye fixations between depletion and control conditions, and these differences are not captured by eye movements.

In general, future studies should focus on testing the validity of the most frequently used ego depletion tasks. How long a self-control task needs to last to successfully deplete self-control strength should be analyzed. There is no general agreement on this matter as, for instance, the Stroop task contains only 50 trials in some experiments [35] and 250 words in other studies [36]. These differences might also be a reason for the heterogeneous findings for ego depletion (however, that different types of standardization of the manipulation did not produce meaningful effects in this experiment shows that there may be some tolerance in terms of designing the methods). In the same vein, it is unclear how long it takes to replenish depleted self-control strength [12].

Future studies should also adopt the other prominent self-control theories to test the validity of the strength model of self-control. For instance, implicit theories about willpower should be assessed: Job and colleagues [35] have repeatedly demonstrated that the ego depletion effect is primarily found in individuals who have a limited implicit theory about willpower, while the performance of individuals who have a non-limited implicit theory about willpower does not suffer under ego depletion.

## Conclusion

The present study found only moderate empirical evidence for the assumption that the attention control video requires self-control strength. It seems promising to adopt objective measures of self-control (e.g. eye tracking) in order to dig deeper into the *true* nature of the ego depletion effect. Future studies should also focus on investigating whether there is actually something like a limited self-control resource. Previous research suggested that glucose seems to be the driving physiological force of self-control performance [37], however these findings have been difficult to replicate [38,39]. To conclude, currently there seem to be more questions than answers in regard to the ego depletion effect and more rigorous research adopting appropriate research designs is highly necessary.

## Supporting information

S1 File Study protocol. dx.doi.org/10.17504/protocols.io.uj3euqn.
(PDF)

S2 File Descriptive statistics: Means and Standard Deviations for condition by type of standardization of the transcription task. (PDF)
