# Supplementary material for: Testing the validity of the attention control video: An eye-tracking approach of the ego depletion effect
Source: PLoS One. 2019 Jan 22;14(1):e0211181. doi: 10.1371/journal.pone.0211181 (PMC6342314; doi:10.1371/journal.pone.0211181)
Supplement: S1 File — dx.doi.org/10.17504/protocols.io.uj3euqn. (PDF) [file pone.0211181.s001.pdf]

Article title: Testing the validity of the attention control video: an eye-tracking approach

Author names: Chris Englert, Dennis Koroma, Alex Bertrams & Corinna S. Martarelli

Corresponding author: Chris Englert, University of Bern, Institute of Educational Science, Department of Educational Psychology, Fabrikstrasse 8, 3012 Bern, Switzerland, Phone: +41 (0)31 631 8275, Email: christoph.englert@edu.unibe.ch

Fragebogen

1 Bedingung

Versuchspersonennummer:

Bedingung:

○ Ego Depletion ○ Kein Ego Depletion

2 Standardseite

Liebe Studienteilnehmerin, lieber Studienteilnehmer,

vielen Dank für Ihre Teilnahmebereitschaft an der Studie „Selbstkontrolle und Aufmerksamkeit“. Sie werden im Verlauf der Erhebung gebeten, verschiedene kognitive Aufgaben zu bearbeiten und Fragebögen auszufüllen. Dabei gibt es keine richtigen oder falschen Antworten, bedeutsam ist Ihre persönliche Einschätzung. Zudem werden Ihre Daten vertraulich behandelt und Sie bleiben vollständig anonym. Bitte folgen Sie den Instruktionen auf dem Bildschirm und wenden Sie sich per Handzeichen an die Versuchsleitung, wenn Sie auf dem Bildschirm dazu aufgefordert werden.

Vielen Dank

3 Selbstkontrolle

Bitte kreuzen Sie im Folgenden jeweils eine Zahl zwischen 1 (völlig unzutreffend) und 5 (trifft ganz genau zu) an um auszudrücken, wie sehr die betreffende Aussage Ihrer Meinung nach im Allgemeinen, also üblicherweise, auf Sie zutrifft:

	1	2	3	4	5
Ich bin gut darin, Versuchungen zu widerstehen.	○	○	○	○	○
Es fällt mir schwer, schlechte Gewohnheiten abzulegen.	○	○	○	○	○
Ich bin faul.	○	○	○	○	○
Ich sage unangemessene Dinge.	○	○	○	○	○
Ich tue manchmal Dinge, die schlecht für mich sind, wenn sie mir Spaß machen.	○	○	○	○	○
Ich wünschte, ich hätte mehr Selbstdisziplin.	○	○	○	○	○
Angenehme Aktivitäten und Vergnügen hindern mich manchmal daran, meine Arbeit zu machen.	○	○	○	○	○
Es fällt mir schwer, mich zu konzentrieren.	○	○	○	○	○
Ich kann effektiv auf langfristige Ziele hinarbeiten.	○	○	○	○	○
Manchmal kann ich mich selbst nicht daran hindern, etwas zu tun, obwohl ich weiß, dass es falsch ist.	○	○	○	○	○
Ich handle oft, ohne alle Alternativen durchdacht zu haben.	○	○	○	○	○
Ich lehne Dinge ab, die schlecht für mich sind.	○	○	○	○	○
Andere würden sagen, dass ich eine eiserne Selbstdisziplin habe.	○	○	○	○	○

4.1 Ego Depletion

Liebe Versuchsteilnehmerin, lieber Versuchsteilnehmer

nun folgt eine Aufmerksamkeitsaufgabe: Schreiben Sie auf das beiliegende Blatt einen Text ab, der gleich auf dem Bildschirm erscheinen wird. Schreiben Sie möglichst schnell, aber vermeiden Sie Leichtsinnsfehler. Das Besondere an dieser Aufgabe ist, dass Sie immer die Buchstaben „e“ und „n“ auslassen.

Beispielsweise wird der Beginn des Textes wie folgt abgeschrieben: „Als hmalig Rsidzstadt dr historisch Kurpfalz bildt Mahim bis hut...“. Bearbeiten Sie den gesamten Text auf diese Weise. Wenden Sie sich nun nochmals an die Versuchsleitung.

5.1 No Ego Depletion

Liebe Versuchsteilnehmerin, lieber Versuchsteilnehmer

nun folgt eine Aufmerksamkeitsaufgabe: Schreiben Sie auf das beiliegende Blatt einen Text ab, der gleich auf dem Bildschirm erscheinen wird. Schreiben Sie möglichst schnell, aber vermeiden Sie Leichtsinnsfehler. Wenden Sie sich nun nochmals an die Versuchsleitung.

6 Text

Als ehemalige Residenzstadt der historischen Kurpfalz bildet Mannheim bis heute das wirtschaftliche und kulturelle Zentrum der Region. An seine kulturelle Blütezeit im 18. Jahrhundert konnte es zwar nicht wieder anknüpfen, hat sich aber in seiner wechselvollen Geschichte immerhin um die Erfindung des Zweirads, Automobils und der Landmaschinen verdient gemacht.

Das Dorf Mannenheim (Heim des Manno) wird im Jahre 766 erstmals im Lorscher Codex urkundlich erwähnt. Lange Jahre war es ein kleines unbedeutendes Fischerdorf. 1284 fiel Mannheim an den Pfalzgrafen bei Rhein aus dem Hause Wittelsbach. Dagegen wird Neckarau bereits 368 geschichtskundig. Es befand sich der Burgus des Kastells Alta Ripa (Altrip) auf heutiger Neckarauer Gemarkung. 771 wird das Dorf Hermsheim erstmals im Lorscher Codex erwähnt. 1212 schenkte Kaiser Friedrich II. Neckarau dem Bischof von Worms. 1294-1365 veränderte der Neckar seine Mündung in den Rhein, daher wurde das Dorf Hermsheim nach Neckarau verlegt.

7 Fragen_Abschreibaufgabe

Die nun folgenden Fragen beziehen sich auf die soeben durchgeführte Abschreibaufgabe. Markieren Sie bitte das für Sie Zutreffende:

	überhaupt nicht	ein wenig	ziemlich	sehr
Wie mental erschöpft fühlen Sie sich gerade?	○	○	○	○
Wie anstrengend fanden Sie die Aufgabe?	○	○	○	○
Wie schwierig war es für Sie, den Instruktionen zu folgen?	○	○	○	○
Inwieweit mussten Sie Ihre Schreibgewohnheiten regulieren?	○	○	○	○
Denken Sie, dass Sie bei der Abschreibaufgabe gut abgeschnitten haben?	○	○	○	○

8 Emotionen

Geben Sie nun bitte an, wie sehr die jeweilige Emotion in diesem Moment auf Sie zutrifft.

	gar nicht	ein bisschen	einigermassen	erheblich	äusserst
interessiert	○	○	○	○	○
bekümmert	○	○	○	○	○
freudig erregt	○	○	○	○	○
verärgert	○	○	○	○	○
stark	○	○	○	○	○
schuldig	○	○	○	○	○
erschrocken	○	○	○	○	○
feindselig	○	○	○	○	○
begeistert	○	○	○	○	○
stolz	○	○	○	○	○
gereizt	○	○	○	○	○
wach	○	○	○	○	○
beschämt	○	○	○	○	○
angeregt	○	○	○	○	○
nervös	○	○	○	○	○
entschlossen	○	○	○	○	○
aufmerksam	○	○	○	○	○
durcheinander	○	○	○	○	○
aktiv	○	○	○	○	○
ängstlich	○	○	○	○	○

9 Versuchsleitung

Wenden Sie sich nun bitte an die Versuchsleitung

10 Video

Sie werden gleich ein sechsminütiges Video ohne Ton sehen. In diesem Video ist eine Frau zu sehen, die von einer Person außerhalb der Kameraperspektive interviewt wird.
Wichtig: Lesen Sie bitte nicht irgendwelche Wörter, die auf dem Bildschirm erscheinen! Richten Sie Ihren Blick ausschließlich auf die Frau in dem Video und nicht auf erscheinende Wörter. Wenn Sie merken, dass Sie doch auf ein Wort gesehen haben, richten Sie Ihren Blick und Ihre Aufmerksamkeit bitte umgehend wieder auf die Frau und vermeiden Sie weiterhin, auf die Wörter zu sehen.
Während des Videos werden wir Ihr Blickverhalten aufzeichnen. Wenden Sie sich nun bitte an die Versuchsleitung.

11 Fragen_Video

Die nun folgenden Fragen beziehen sich auf das soeben dargestellte Video. Markieren Sie bitte das für Sie Zutreffende (1= gar nicht; 7= sehr):

	1	2	3	4	5	6	7
Wie sehr haben Sie es unterdrückt, zu den im Video erscheinenden Wörter hinzusehen?	○	○	○	○	○	○	○
Wie anstrengend fanden Sie es, das Video anzusehen?	○	○	○	○	○	○	○
Wie sympathisch erschien Ihnen die Frau in dem Video?	○	○	○	○	○	○	○
Wie aufgeregt erschien Ihnen die Frau in dem Video?	○	○	○	○	○	○	○
Wie aufmerksam erschien Ihnen die Frau in dem Video?	○	○	○	○	○	○	○
Wie introvertiert erschien Ihnen die Frau in dem Video?	○	○	○	○	○	○	○
Wie interessiert erschien Ihnen die Frau in dem Video?	○	○	○	○	○	○	○
Wie engagiert erschien Ihnen die Frau in dem Video?	○	○	○	○	○	○	○

12 Meinung

1. Beschreiben Sie bitte mit Ihren Worten und aus Ihrer Sicht, was wir untersuchen:

2. Möglicherweise haben Sie schon im Vorfeld etwas zur Untersuchung gehört. Ist dies der Fall, ist es in Ordnung, aber wir müssen dies berücksichtigen. Haben Sie etwas gehört?

○ Ja

○ Nein

Falls ja, was haben Sie gehört?

13 Standardseite

Die folgenden Angaben dienen statistischen Zwecken. Sie bleiben **anonym**.

14 demographische Angaben

Was haben Sie für ein Geschlecht?

○ weiblich ○ männlich

Alter:

Ihre Nationalität:

Ihr Beruf:

Sprechen Sie fliessend deutsch?

○ ja ○ nein

16 Endseite

Herzlichen Dank für Ihre Teilnahme! Wenden Sie sich nun bitte an die Versuchsleitung.
