# Supplementary material for: Testing the validity of the attention control video: An eye-tracking approach of the ego depletion effect
Source: PLoS One. 2019 Jan 22;14(1):e0211181. doi: 10.1371/journal.pone.0211181 (PMC6342314; doi:10.1371/journal.pone.0211181)
Supplement: S2 File — (PDF) [file pone.0211181.s002.pdf]

Article title: Testing the validity of the attention control video: an eye-tracking approach

Author names: Chris Englert, Dennis Koroma, Alex Bertrams & Corinna S. Martarelli

Corresponding author: Chris Englert, University of Bern, Institute of Educational Science, Department of Educational Psychology, Fabrikstrasse 8, 3012 Bern, Switzerland, Phone: +41 (0)31 631 8275, Email: christoph.englert@edu.unibe.ch

Descriptive Statistics: Means and Standard Deviations for Condition by Type of Standardization of the Transcription Task.

	**Depletion – fixed length of time** ***M (SD), n = 29***	**Depletion – fixed number of words** ***M (SD), n = 29***	**Control – fixed length of time** ***M (SD), n = 27***	**Control – fixed number of words** ***M (SD), n = 27***
SCS-K-D	3.30 (0.49)	2.98 (0.49)	3.30 (0.54)	3.22 (0.59)
Manipulation Check Transcription task	2.36 (0.47)	2.39 (0.54)	1.77 (0.39)	1.70 (0.43)
PANAS positive	2.93 (0.69)	2.89 (0.70)	2.77 (0.80)	2.71 (0.59)
PANAS negative	1.29 (0.27)	1.34 (0.27)	1.29 (0.29)	1.17 (0.20)
Report on gaze behavior Item 1	5.00 (1.54)	4.48 (2.01)	5.24 (1.55)	4.63 (1.55)
Report on gaze behavior Item 2	4.83 (1.49)	4.41 (1.53)	4.45 (1.40)	4.56 (1.50)

Note. SCS-K-D = German short version of the Self-Control Scale. Manipulation Check Transcription Task = four-item manipulation check (e.g., "How strongly did you have to regulate your writing habits?"). PANAS positive = German version of the Positive and Negative Affect Schedule – positive affect. PANAS negative = German version of the Positive and Negative Affect Schedule – negative affect. Report on gaze behavior Item 1 = "How strongly did you have to force yourself to not pay attention to the words". Report on gaze behavior Item 2 = "How difficult was it to not pay any attention to the words?"
